# Exclusive breastfeeding practices and challenges in Nigeria, Sub-Saharan Africa: an integrative review

**DOI:** 10.1186/s13006-026-00810-3

**Published:** 2026-02-03

**Authors:** Ugochi Felicitas Oputa-Uzoukwu, Friday Ilop Joseph

**Affiliations:** 1 School of Criminology, Sociology, and Policing, University of Hull, Hull, UK; 2 Department of Pediatrics, University College Hospital, Ibadan, Oyo State, Nigeria

**Keywords:** exclusive breastfeeding, integrative review, cultural barriers, children, Nigeria

## Abstract

**Background:**

Exclusive breastfeeding (EBF) remains a key public health strategy for improving infant and maternal outcomes. In Nigeria, despite policy commitments, EBF practice rates remain inconsistent and highly context-dependent. This integrative review assesses the prevalence and patterns of EBF in Nigeria, examines the socio-demographic and systemic factors influencing practice, identifies barriers and enablers, and evaluates existing interventions to promote EBF.

**Methods:**

Nineteen peer-reviewed primary studies, published between 2013 and 2024, were systematically identified across six geopolitical zones. Data were extracted from PubMed, ScienceDirect, and the University of Hull library database using a PRISMA search strategy, and synthesis followed a deductive coding framework, drawing on qualitative and quantitative findings. The theoretical frameworks for this review were the Socio-Ecological Model (SEM) and the Health Belief Model (HBM), which provide insights into the influence of individual, interpersonal, community, and societal factors on exclusive breastfeeding practices in Nigeria.

**Results:**

Analysis of 19 studies in Nigeria revealed that the percentage of women that exclusively breastfed their infant from birth to six months averaged 46.4% (range 12.5%–73.8%), while early initiation within one hour of delivery averaged 57.3%. Despite high awareness, a significant “knowledge-practice gap” exists due to complex socio-contextual and maternal factors. Higher EBF rates correlate with maternal age (31+), tertiary education, and hospital deliveries. Conversely, employment in the formal sector often hinders EBF due to short maternity leave. Primary barriers include cultural myths (viewing colostrum as “dirty” or breastmilk as “insufficient” without water) and lack of spousal/family support. Facilitators include healthcare engagement (ANC/PNC) and high maternal self-efficacy. The review also identified gaps in research, including the limited inclusion of fathers, informal caregivers, and mothers in displaced or rural communities.

**Conclusion:**

This review advances the literature by offering a context-sensitive, multi-dimensional interpretation of EBF practices in Nigeria. It highlights the need for equity-focused policies and interventions that move beyond individual awareness to address structural enablers of maternal capabilities.

**Supplementary Information:**

The online version contains supplementary material available at 10.1186/s13006-026-00810-3.

## Background

Every infant and child has the right to good nutrition, as stated in the “Convention on the Rights of the Child [1]. In 2024, the WHO and UNICEF estimated that 150.2 million children were too short for their age, 42.8 million were too thin for their height, and 35.5 million were overweight or obese [2]. Poor child nutrition is linked to approximately 50% of child mortality. Improving nutrition could save over 820,000 lives annually among children under five if all children aged 0–23 months received optimal breastfeeding. WHO defined optimal breastfeeding as initiating breastfeeding within the first hour after birth and exclusively breastfeeding for the first six months of life, without introducing any other foods or liquids, including water. From six months onward, children should start consuming safe and adequate complementary foods while continuing to breastfeed for up to two years or beyond [3].

Exclusive breastfeeding is a highly cost-effective method to enhance child health and survival. It supplies all the necessary energy and nutrients for infants in the first few months of life. During the latter half of the first year, it still provides up to half or more of a child’s nutritional needs, and about one-third in the second year [3, 4]. Human milk typically offers around 67 kcal per 100 mL, mainly from 4.2 g of fat and 7.0 g of carbohydrates (primarily lactose), along with 1.3 g of easily digestible protein. Importantly, breast milk also contains non-nutritional components, such as antibodies and Human Milk Oligosaccharides (HMOs), which are essential for strengthening the infant’s immune system and gut health [5]. Breastfed children tend to score higher on intelligence tests, are less likely to become overweight or obese, and have a lower risk of developing diabetes later in life. Furthermore, women who breastfeed face a decreased risk of breast and ovarian cancers [3, 4].

Contrary to the WHO set target of 50% of infants being exclusively breastfed for the first 6 months of life by 2025, only 38% of infants worldwide are exclusively breastfed within the first six months of life. There is a strong connection between optimal breastfeeding and the reduction of diarrhea and pneumonia, which are the leading causes of death among children under five in developing countries [6, 7]. Exclusive breastfeeding can reduce the rate of pneumonia among young infants by 15 to 23%. Globally, in 2021, the number of childhood deaths due to diarrheal diseases linked to suboptimal and non-exclusive breastfeeding was 63,133 and 54,770, respectively [4]. Western Sub-Saharan Africa, South Asia, and Eastern Sub-Saharan Africa ranked as the top three regions concerning suboptimal/non-exclusive attributed deaths [8].

Globally, the promotion of optimal breastfeeding faces numerous socio-cultural barriers, along with poor policy design and weak program implementation. Evidence suggests that many women lack access to a supportive environment for breastfeeding, partly due to a lack of social support, inadequate funding, poor enforcement and monitoring of laws, and limited institutional capacity to promote and safeguard optimal breastfeeding practices [9].

In Nigeria, infant mortality rates are alarmingly high, with one in 25 infants dying within their first month and one in 16 not surviving their first year [10]. This highlights the urgent importance of adequate nutrition and care during the critical first 1000 days of life. The 2024 Nigeria Demographic and Health Survey (NDHS) notes that only 36% of newborns are breastfed within the first hour, and 29% of children under six months are exclusively breastfed.

In 2016, Nigeria recorded 22,371 deaths from diarrhea among children under five, largely linked to poor breastfeeding practices [8]. These practices accounted for about 56.5% of diarrhea deaths in the late neonatal period, 39.0% during post-neonatal and infancy stages, and 22.8% in children under five [8]. Additionally, the estimated Disability-Adjusted Life Years (DALYs) due to diarrhea from suboptimal breastfeeding amounted to 1.9 million among children under five [4, 8].

Non-exclusive breastfeeding (NEBF) increases infant mortality due to higher infection risk and malnutrition. It reduces infants’ intake of essential antibodies and HMOs, weakening their immune defense [5, 11]. Introducing non-sterile foods can cause contamination, while NEBF can lead to undernutrition and decreased milk supply from the mother, complicating breastfeeding [11]. Nigeria ranks first in deaths among children under five due to suboptimal and non-exclusive breastfeeding, followed by India and Chad [4]. Given the critical role optimal breastfeeding hold for the survival of under five children in Nigeria, this integrative review aims to examine the prevalence, characteristics, and various influences—such as individual, cultural, socio-economic, and healthcare factors—on exclusive breastfeeding (EBF), with the goal of developing effective strategies and policies to increase EBF rates and enhance public health outcomes. The main objectives are to address the following questions: “What is the prevalence of exclusive breastfeeding (EBF) and its socio-demographic characteristics in Nigeria?” “What factors influence the decision to initiate and continue exclusive breastfeeding as recommended by WHO?” and “What interventions are available in Nigeria to promote exclusive breastfeeding?”.

## Methods

This study employed an integrative literature review (ILR) to critically synthesize existing research on exclusive breastfeeding (EBF) in Nigeria, acknowledging the topic’s evolving nature. Rather than attempting to include all publications exhaustively, the integrative literature review facilitates the critical evaluation and creative integration of diverse perspectives to generate new theoretical understandings [12, 13]. This approach aligns with the view that for broad, interdisciplinary topics, a strict systematic review may be impractical, making a more flexible, narrative method preferable [13]. By incorporating both quantitative data, such as prevalence rates, and qualitative insights into participants’ experiences, the review presents a comprehensive and multifaceted view of the factors influencing EBF practices in the region, thereby informing the development of tailored policies and interventions. Through this synthesis, the study not only addresses specific research questions but also reconceptualizes the topic, considering emerging patterns and challenges [12, 14].

**Fig. 1:**
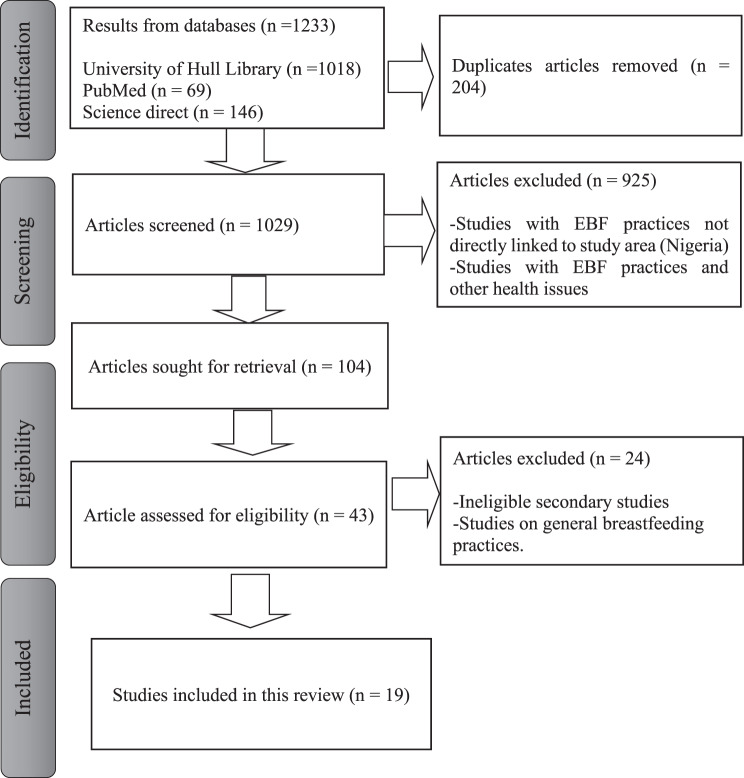
PRISMA flow diagram showing the identification, screening, eligibility, and inclusion of studies in the review

The data are from three databases: PubMed, ScienceDirect, and the University of Hull library, using search terms such as “exclusive breastfeeding practices and challenges in Nigeria” and “breastfeeding practices in Nigeria and Sub-Saharan Africa.” The searched databases, as illustrated in [Fig. 1] (PRISMA flow diagram), identified 1233 results; 204 duplicates were removed, and 1029 studies were screened based on titles and abstracts. Forty-three studies were assessed for eligibility, and only 19 studies met eligibility criteria for review.

The study systematically sourced data from research conducted and published between January 2013 to December 2024, aligning search criteria with research objectives and considering research period, publication date, methodology, and geographic scope. The screening process scrutinized abstracts for relevance to ensure they met the inclusion and exclusion criteria. The inclusion criteria were studies that used primary data involving women of childbearing age, mothers, healthcare workers, and family members engaged in childcare, while exclusion criteria targeted studies of secondary data review and analysis, non-English studies, those predating 2013, and studies focusing on health issues unrelated to exclusive breastfeeding.

For quality appraisal, this review adopted the approach of an “own-assessment checklist” [15], arguing that critical appraisal methods may not reveal the validity of a study because several checklist limitations may affect research conduct and reporting in a journal. The primary focus here is to assess whether the reviewed studies provide accounts of the study objectives, design, participants’ selection, method of data collection, analysis, and ethical considerations. Studies reveal that the structured appraisal methods employed by reviewers are insufficient criteria for excluding a paper based on quality [16]; hence, including all research helps minimize a possible source of bias rather than excluding it based only on quality [17]. The Study date and the adopted quality appraisal questions are listed in Table 1, and the detailed responses are included in Table 2 below.

**Table 1:** Adopted quality appraisal checklist for reporting reviewed studies

Question	Definition and assessment: Yes/No/Unclear
The study design and approach reasons	Yes (If the choice of study design was given and explained) if it states, e.g. “a case study approach was used because …”,“interviews were used because …” No if paper does not specify question and study design
The selection of participants	Yes, if paper describes selection explicitly as e.g. purposive, convenience, theoretical etc. No if just details of participants are given
Methods of data collection	Yes, if details of data collection method are given e.g. piloting; topic guides for interviews; number of items in a survey; use of open or closed items; validation; etc. No if just states “focus group”, “interview” or “questionnaire”
Methods of analysis	Yes, if details of analysis are given, e.g. transcription, form of analysis (with reference), etc. No if just states “content analysis” or data were “analyzed”

**Table 2:** Responses to the adopted quality appraisal questions

	Author (Year)	Study Date/Year	The study design and approach (Yes, No)	The selection of participants (Yes, No)	Methods of data collection (Yes, No)	Methods of data analysis (Yes, No)
1	Adamu et al. (2022) [18]	Jan to Sept, 2016	Yes (Reasons not stated)	Yes	Yes	Yes
2	Aliyu et al. (2019) [19]	Oct to Dec 2017	Yes (Reasons not stated)	Yes	Yes	Yes
3	Akadri & Odelola (2020) [20]	No data	Yes	Yes	Yes	Yes
4	Yakubu et al. (2023) [21]	No data	Yes	Yes	Yes	Yes
5	Anaba et al. (2022) [22]	Sept to Oct 2019	Yes	Yes	Yes	Yes
6	Anyanwu et al. (2014) [23]	No data	Yes	Yes	Yes	Yes
7	Atimati & Adam (2020) [24]	May 2016 to Jan 2017	Yes	Yes	Yes	Yes
8	Bisi-Onyemaechi et al. (2017) [25]	No data	Yes	Yes	Yes	Yes
9	Mohammed & Aliyu (2021) [26]	No data	Yes	No	Yes	Yes
10	Odu et al. (2016) [27]	No data	Yes	Yes	Yes	Yes
11	Okoroiwu et al. (2021) [28]	No data	Yes	Yes	Yes	Yes
12	Olasinde et al. (2021) [29]	May to Jun 2020	Yes	Yes	Yes	Yes
13	Balogun et al. (2017) [30]	No data	Yes	Yes	Yes	Yes
14	Elegbua et al. (2023) [31]	No data	Yes	Yes	Yes	Yes
15	Amat Camacho et al. (2023) [32]	Mar 2022	Yes	Yes	Yes	Yes
16	Joseph & Earland (2019) [33]	Jun to Jul 2016	Yes	Yes	Yes	Yes
17	Anazonwu et al. (2018) [34]	No data	Yes	Yes	Yes	Yes
18	Ugboaja et al. (2013) [35]	No data	Yes	Yes	Yes	Yes
19	Ogundairo et al. (2024) [36]	Jun 2020 to Dec 2021	Yes	No	Yes	Yes

The quality appraisal checklist used in this study was adapted from [15] and refined to align with the research objectives and context.

Given the high rate of published papers on breastfeeding practices in Nigeria, the primary purpose of this review is to draw insights from both what is known and what is unknown about the topic and to provide direction on prominent, underexplored areas for further research.

This study adhered to ethical research practices by utilizing publicly accessible databases, with emphasis on research integrity, transparency, confidentiality, and appropriate citation of sources. Informed consent was not required as the study used secondary data from publicly available sources. Ethical clearance was obtained from the University of Hull’s Faculty Research Ethics Committee in accordance with institutional ethical standards.

### Description of extracted data and analysis

Data is structured on 19 reviewed studies conducted across the 36 states and the capital of Nigeria, made up of six geo-political zones, namely the North Central (NC), North-West (NW), North-East (NE), South-East (SE), South-West (SW), and South-South regions (SS). The summary of the studies is found in Supplementary Table 1, listed in alphabetical order, comprising geo-political zones, study area, setting, study methodology, socio-demographic characteristics of respondents, and study findings including EBF practice rate, Early initiation, skin-to-skin contact after birth, and continued breastfeeding up to 2 years. The study locations are as follows: NW - Sokoto (2) Kebbi (2), Zamfara, Katsina, Kano, Kaduna; NE - Borno; NC - Abuja (FCT); SW - Ogun, Lagos, Osun, Oyo (2); SE - Enugu (2), Ebonyi, Anambra; and SS – Edo and Rivers state. The study areas were Urban = 8, Urban & Rural = 3, Semi-Urban = 4, Rural = 2, Non-specific wards = 1, Humanitarian = 1. Participants were recruited in various study settings, including THF = 7, THF & SHF = 1, PHCs = 3, Community and markets = 5, LGAs = 1, non-specific wards = 1, and MSN project Centers = 1. Fourteen papers adopted a quantitative study method: Cross-sectional Descriptive = 12 [18–29] and Comparative cross sectional = 2 [30, 31], Qualitative study methods were two [32, 33], Mixed method were two [34, 35] and one Quasi-experimental study [36] which was a Longitudinal cohort study of two groups carried out mid pregnancy to 6 months post-partum. Across the 19 studies, a total of 7573 participants, including mothers and infant caregivers, were included. Six studies were conducted and published between 2013 and 2018, while 13 studies were conducted between 2019 and 2024.

Data from these studies were manually reviewed and extracted into an evidence table, coded using a deductive approach, analyzed and synthesized with the aid of a MS Word pre-defined codebook and Excel spreadsheet. Drawing on Saldaña’s coding framework [37], our analysis integrated prevalence rates, statistical findings, and qualitative data to interpret recurring patterns and connections in exclusive breastfeeding (EBF) practices across Nigeria.

### Theoretical framework

The research is guided by Urie Bronfenbrenner’s Socio-Ecological Model (SEM), which was developed in the 1970s and later expanded in the 1980s, and the Health Belief Model (HBM), established by Hochbaum and Rosenstock in the 1950s. These theoretical frameworks provide insights into the influence of individual, interpersonal, community, and societal factors on health behaviors, offering a comprehensive understanding of the complexities of attitudes and behaviors for effective public health interventions [38–40].

**Fig. 2:**
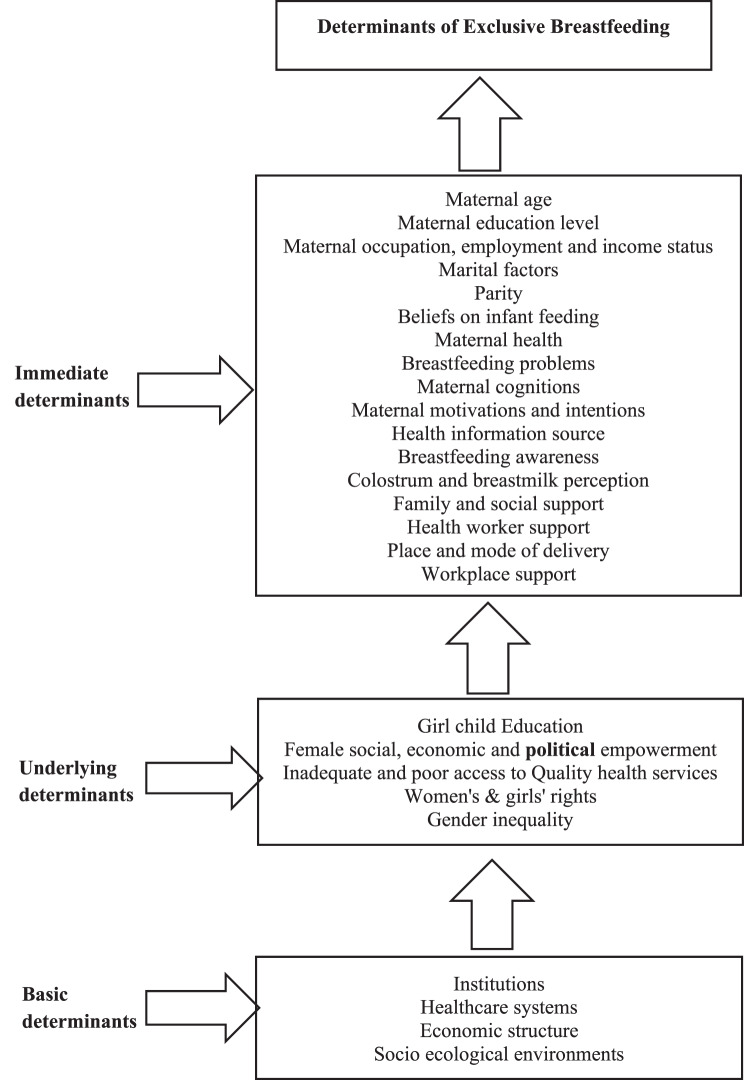
Conceptual framework illustrating the determinants of exclusive breastfeeding in Nigeria, including individual, familial, and sociocultural factors

This study further proposes a causal conceptual framework building on the foundational theories of the Socio-Ecological Model (SEM) and the Health Belief Model (HBM), to explore the range of factors that influence exclusive breastfeeding (EBF) practices. These theories highlight how individual beliefs, social influences, and broader systemic conditions interact to shape maternal health behavior. Informed by these perspectives, the framework groups the potential determinants of EBF into three main categories: Immediate, Underlying, and Basic determinants (see Fig. 2). This structure reflects the complex realities mothers face in Nigerian communities and helps to organize the social, cultural, and structural influences that support or hinder EBF.

## Results

**Fig. 3:**
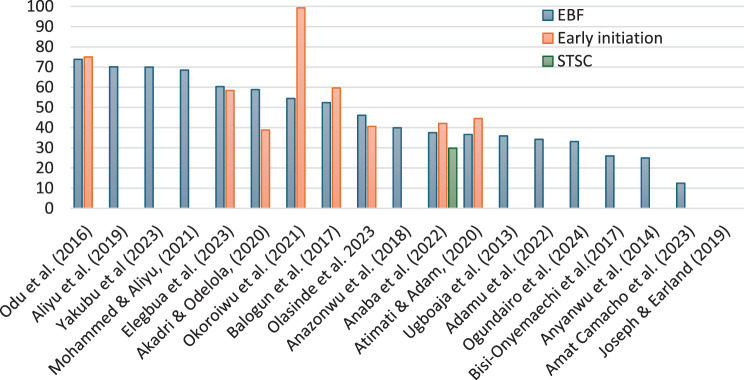
Prevalence of key breastfeeding practices across 19 studies.*EBF = exclusive breastfeeding; EI = early initiation of breastfeeding; STSC = skin-to-skin contact at birth*

An analysis of 19 studies highlights the actual prevalence rates of key breastfeeding practices for infants aged 0–6 months. The percentage of women that exclusively breastfed their infant from birth to six months averaged 46.4% (range 12.5%–73.8%). Early breastfeeding initiation within the first hour after birth was reported in 9 studies, showing prevalence rates from 38.8% to 99.3% with a mean of 57.3%. Only one study reported immediate skin-to-skin contact at birth, with a prevalence of 29.8% [22]. One study reported both EBF and early initiation as ‘low’ without providing specific percentages [33]. Detailed prevalence data for all studies are presented in Fig. 3 and Supplementary Table 2.

**Fig. 4:**
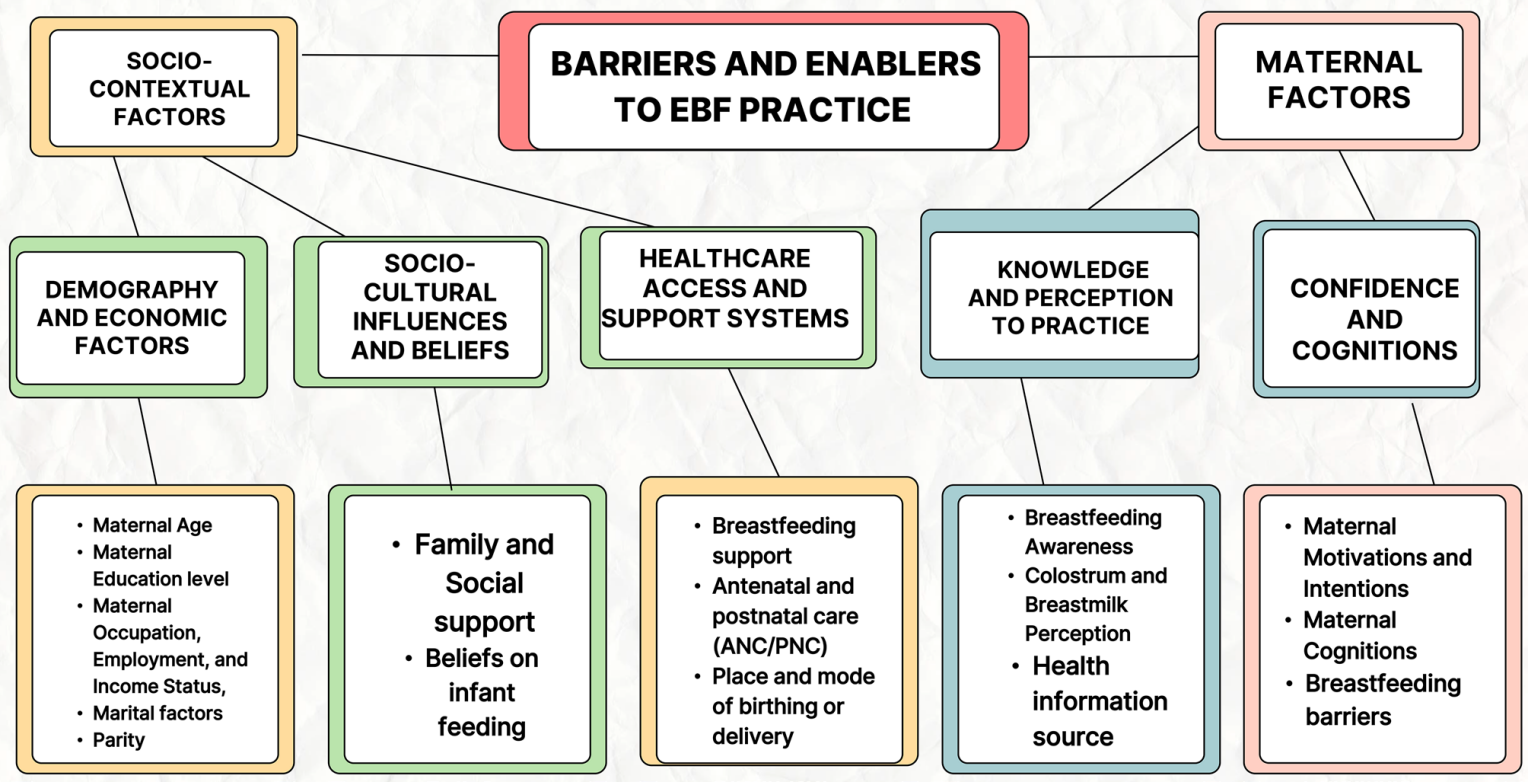
Thematic summary of barriers and enablers of exclusive breastfeeding in Nigeria, highlighting major influencing factors at multiple levels

Using thematic content analysis, the results identified two primary themes: Socio-Contextual factors and Maternal factors. These themes were developed from five sub-themes and sixteen categories. The sub-themes are: (1) Socio-demography and economic Factors; (2) Socio-cultural influences and beliefs; (3) Healthcare access and support systems; (4) Maternal knowledge and perceptions about practice; and (5) Maternal confidence, cognitions, and barriers to EBF practice. These themes are summarized in Fig. 4, showing the Barriers and Enabling factors for EBF practice in Nigeria.

## Socio-contextual factor

### Socio-demography and economic factors: prevalence and patterns

This theme includes factors that positively or negatively affects EBF practices. Essential elements consist of maternal age, education, occupation and income, parity, and marital status.

#### Maternal age

There is a significant link between breastfeeding self-efficacy and maternal age [21, 24, 29, 35, 36]. Higher exclusive breastfeeding (EBF) rates were observed among older mothers (aged 31 and above) compared to those aged 30 and below [24, 29]. Additionally, delayed breastfeeding initiation was more common in mothers aged 20 years and below. However, a few studies have noted a decline in EBF practice with increasing maternal age in the 20–29 age group [21, 35].

#### Maternal education levels

Maternal level of education showed a strong link with exclusive breastfeeding (EBF) practice among mothers with higher education levels. Mothers with tertiary education had significantly better knowledge, practice, and acceptance of EBF, and likely to practice EBF [24, 26]. Other studies indicate that mothers with more formal education are more likely to practice EBF [18, 35, 36], with one study reporting a significant effect (*p* = 0.003) [21]. However, one study found that while EBF was more common among younger women (under 35) and those with more than primary level of education, maternal age and education level were not significant determinants of EBF practice [25].

#### Maternal occupation, employment and income status

Maternal occupation, employment and income status were identified in 11 of the 19 reviewed studies as influencing exclusive breastfeeding practices. Several studies highlighted maternal occupation as a key factor, with the type of job impacting both the start and continuation of breastfeeding. Jobs in the formal sector, such as health workers, teachers, and business owners, were more positively linked to EBF [21, 26]. Similarly, maternal occupation predicted breastfeeding self-efficacy and initiation [36]. Conversely, Civil servants faced high work-related pressure, with 19.9% and 61.8% of mothers respectively citing job demands as reasons for stopping EBF [23, 34]. Employment-related issues, especially short maternity leave and early return to work, were often identified as obstacles to exclusive breastfeeding [18, 19, 23, 25, 29, 30, 34] as mothers returning to work soon after childbirth faced significant challenges in maintaining EBF. In one study, 17.7% of employed mothers stopped EBF due to limited maternity leave [18]. Another study, reported that 88.5% of mothers who did not return to work early successfully practiced EBF, whereas only 11.5% did not [25]. Therefore, early re-entry into the workforce was a common barrier [19, 29, 30]. Income level and employment status influence EBF behaviors. The review showed that many mothers who were artisans or traders earned less than the $20 monthly minimum wage and had lower breastfeeding self-efficacy [36]. Conversely, employment isn’t always a negative factor as mothers in paid roles, including those in low-income households and on-site jobs, showed higher rates of early breastfeeding initiation [22]. Additionally, some evidence indicates that unemployment can support EBF. One study noted that mothers not in paid employment practiced EBF more often than civil servants, with a significant difference (*p* = 0.007) [18].

#### Marital factors

Marital dynamics like polygyny, early marriage, and domestic violence as barriers to EBF, noting that spouses often influence decisions to start or continue breastfeeding [32]. Meanwhile, one study observed that marital status positively affects EBF practice [36]. These opposing findings emphasize the complexity of marital roles and suggest a need for further investigation.

#### Parity

This variable is important, especially since the reviewed studies include both primiparous and multiparous mothers, offering different views on childbearing and infant nutrition challenges. Notably, only one study reported that parity influenced early breastfeeding initiation, with some first-time mothers delaying initiation due to traditional practices [33]. Meanwhile, a mother’s ability to breastfeed may change over time, often declining with age and the number of births, as they could breastfeed their first children but faced difficulties with subsequent ones [32]. Across 11 studies reporting participants’ parity, only three studies documented an EBF practice rate of over 40% [20, 26, 29]. This suggests that parity might have a complex, context-dependent impact on exclusive breastfeeding, warranting further research.

### Socio-cultural influences and beliefs

This theme emphasizes the influences of community, family, friends, and beliefs on infant feeding, including personal anxiety, cultural, traditional, and religious practices, which determine infant feeding behaviors.

#### Family and social support

Mothers’ exclusive breastfeeding (EBF) practices are strongly influenced by both internal and external family members, as well as broader social networks. Several studies have identified family support as a positive factor; encouragement from family and friends significantly predicts successful EBF [21, 25, 32]. Similarly, one study emphasized the critical role of spousal support in promoting breastfeeding [27]. Conversely, some research highlights family dynamics as barriers; family approval can support EBF, and the lack of family or social support can hinder it [34]. One study observed that larger families (more than four members) often motivated EBF due to resource constraints, yet mothers also faced pressure from relatives to choose other feeding options [29]. Other studies mentioned direct barriers such as spousal disapproval [22], grandmother’s refusal [35], and the absence of supportive family structures [23]. Additionally, another study pointed out that family members’ decisions and partners’ knowledge of breastfeeding benefits are crucial, with insufficient support ultimately limiting EBF adherence [33].

#### Beliefs on infant feeding

Several studies reveal a common perception that breastmilk alone isn’t enough; many believe infants need water or other liquids for hydration or nutrition. Mothers worry about babies appearing weak, losing weight, or feeling thirsty, leading them to introduce water or herbal mixtures early [18, 21, 30, 32]. While some mothers feared conditions like sunken fontanels or illness, which they thought breastmilk couldn’t prevent [36]. Socio-cultural beliefs, traditional practices, and gender norms significantly influence infant feeding behaviors. Beliefs that infants, especially boys, need more breastfeeding and extra liquids; breastmilk is perceived as inadequate or unnecessary, prompting early use of complementary feeds [22, 34]. One study identified delays in breastfeeding related to infant uvulectomy and the infant’s gender-three days for boys and four for girls [33]. Postpartum practices included herbal treatments applied to the breast for two days and a 40-day period of maternal bathing, which, while culturally important, often delayed early breastfeeding [33]. Although breastfeeding was viewed as a cultural obligation, community norms often disrupted exclusive breastfeeding by delaying initiation for three to four days and giving other liquids, especially during maternal or infant health issues. Traditional healers sometimes advised mothers to stop breastfeeding under conditions like HIV, mastitis, or multiple births, further discouraging or preventing EBF [32]. Additionally, another study reported that fears of infants rejecting other foods later discouraged some mothers from practicing EBF [25]. While culture and illiteracy were linked to adverse EBF outcomes, religion and literacy positively influenced breastfeeding practices [28].

### Healthcare access and support systems

This theme explores how access to healthcare services affects mothers’ health-seeking behaviors related to exclusive breastfeeding (EBF). It covers aspects such as lactation support, the use of health facilities for Antenatal and Postnatal Care (ANC/PNC), and childbirth practices.

#### Breastfeeding support

Supportive interactions with healthcare providers positively affect exclusive breastfeeding (EBF) practices among mothers [22]. Similarly, several studies emphasized the importance of healthcare support through prenatal and postnatal follow-ups [36]. Their intervention study revealed that ongoing healthcare engagement notably extended EBF duration, with 43.2% of mothers in the intervention group maintaining EBF at six months postpartum, compared to 22.9% in the control group, highlighting the benefits of continuous breastfeeding support. However, the success of professional breastfeeding support varies by setting. In a cross-sectional study, mothers visited by lactation experts had a lower exclusive breastfeeding rate (6.4%) than those with no contact with such experts (29%) [25].

#### Antenatal and postnatal care (ANC/PNC) engagement

Of the 19 studies examined, 11 focused on maternal involvement in antenatal and postnatal care (ANC/PNC). Most studies addressed ANC attendance, while two looked at PNC use, and two reported minimal or no engagement with either service. High ANC (97.2%) and PNC (91.7%) attendance positively influenced exclusive breastfeeding (EBF) practices [24, 35]. Similarly, the ANC attendance rate of 98.8%, which significantly impacted EBF outcomes (*p* = 0.0001), mainly through prenatal and postnatal infant feeding education [18].

Mothers’ proximity to health facilities was linked to higher EBF rates, whereas limited access to functional healthcare facilities hindered effective EBF, emphasizing the need for accessible health infrastructure [20, 26, 32]. One study reported that mothers attending ANC four or more times were more likely to initiate breastfeeding early and maintain EBF [22]. Similarly, one study reported that 78.3% of mothers received EBF guidance from health workers during ANC and PNC visits, highlighting the importance of health personnel in providing consistent breastfeeding education through routine ANC and PNC [21, 35]. Though mothers who received ANC from traditional birth attendants (TBAs) also showed early initiation, this subgroup was small and warrants cautious interpretation.

Conversely, one study documented limited ANC engagement, but counselling from healthcare workers and TBAs still promoted early initiation and EBF, despite overall low EBF rates. *A few mothers also recalled receiving advice on colostrum’s benefits during their ANC visits* [33].

#### Place and mode of birthing or delivery

Hospital-based delivery and vaginal birth consistently correlate with better exclusive breastfeeding (EBF) outcomes across multiple studies. The facility type for ANC and delivery showed a significant link to EBF at six months (*p* = 0.001 for ANC; *p* = 0.004 for delivery). Likewise, hospital birth (*p* = 0.004) positively impacted EBF through access to prenatal and postnatal feeding advice [18, 20]. Hospital delivery was also significantly associated with greater breastfeeding knowledge (*p* < 0.05), supporting informed EBF practices [30]. Mothers who delivered in hospitals were more likely to start breastfeeding earlier than those delivering at home [33]. Regarding delivery methods, vaginal delivery is a predictor of breastfeeding self-efficacy, including both initiation and continuation of EBF [30, 33, 36]. One study found that 67.5% of mothers had vaginal deliveries, and 61.3% delivered in hospitals [29]. Both factors were positively linked to EBF practices.

### Maternal knowledge and perception of practice

This theme highlights how maternal knowledge, awareness, and perceptions impact exclusive breastfeeding (EBF) practices. It also shows how understanding breastfeeding guidelines, beliefs about the benefits of colostrum and breastmilk, and sources of health information influence maternal choices.

#### Breastfeeding awareness

Across the reviewed studies, sufficient knowledge and positive perceptions of exclusive breastfeeding (EBF) consistently correlated with better practices. Mothers who understood breastfeeding benefits and accurately knew the guidelines were more likely to start breastfeeding early and maintain EBF [22, 26–28, 31]. Despite widespread awareness, some studies revealed gaps; one study found that 72.2% of mothers knew about EBF, but only 27.8% practiced it, often due to limited understanding of its importance [18]. Another study reported similar issues among female medical practitioners: while 95.1% had general awareness, only 52.1% could define EBF correctly [19]. Also, noted that knowledge gaps among female healthcare workers constrained consistent practice [23].

#### Colostrum and breastmilk perception

Perceptions of colostrum and breastmilk shape behavior. Some mothers and caregivers viewed colostrum as harmful or dirty, leading to its rejection, especially among first-time mothers or those who experienced infant loss. In these cases, breastmilk was often discarded, replaced with herbal mixtures, or breastfeeding was delayed until older relatives or traditional attendants gave their approval [22, 32, 33].

One study highlighted that *“the perceptions of colostrum as bad were a major influence on practice,”* noting that*, “among mothers who had previously experienced neonatal or infant mortality, the child’s death was often attributed to ‘poisonous’ breastmilk. As a result, many of these mothers either refused or were pressured to stop breastfeeding subsequent children”.* [32]

Similarly, another study reported that *“among first-time mothers, colostrum was perceived as ‘dirty,’ with concerns that a baby can contract diseases from it. A few took herbs to make the perceived bad milk good for the baby, while a new mother’s breast milk must be ‘checked’ by a grandmother or a Traditional Birth Attendant before breastfeeding. In subsequent birthing, colostrum was not discarded.”* [33]

In contrast, mothers who understood the nutritional benefits of colostrum were more likely to start exclusive breastfeeding (EBF) early and maintain it. Additionally, women with greater awareness of EBF and its benefits were more inclined to initiate breastfeeding promptly and continue the practice [16, 22].

#### Health information source

Among the studies reviewed, health workers were the most frequently cited source of breastfeeding information. An impressive 92.7% of respondents reported learning about exclusive breastfeeding (EBF) from health professionals [27]. A study found that women who obtained EBF information from mass media were significantly more likely to practice EBF for six months than those who relied on other sources. The study also emphasized the combined role of health workers and mass media in promoting EBF [20]. There is a wider array of information sources, including media, health facilities, NGOs, and community channels [32]. Conversely, in areas where institutional delivery was less common, such as among women delivering at home, breastfeeding guidance mainly came from traditional birth attendants or elder family members like grandmothers.

### Maternal confidence, cognitions, and barriers to EBF practice

This theme reflects the mother’s personal beliefs, motivations, and ability to practice exclusive breastfeeding (EBF). It encompasses exposure to specific interventions, self-confidence, maternal intentions, health status, worries about body image, breastfeeding requirements, lactation challenges, and infant feeding problems.

#### Maternal motivations and intentions

Several studies have shown that targeted interventions effectively promote exclusive breastfeeding (EBF). Community efforts supported by non-governmental organizations like Médecins Sans Frontières (MSF) have positively influenced EBF rates [32]. Studies emphasize the importance of ongoing Social and Behavior Change (SBC) programs in encouraging exclusive breastfeeding (EBF). Visual cues, like pictures of healthy EBF infants, further boost mothers’ motivation to sustain the practice. Additionally, a two-week drama-based intervention before childbirth, combined with postpartum follow-ups, proved effective in supporting continued EBF [22, 36].

Mother’s self-efficacy is vital for maintaining exclusive breastfeeding (EBF). In IDP settings, the lack of trained lactation support staff hampers mothers’ ability to practice EBF effectively, emphasizing the importance of MSF Breastfeeding support in enhancing maternal confidence [32]. Self-motivation plays a crucial role in EBF; mothers with greater confidence tend to engage more actively EBF [21, 22]. A mother’s intention to breastfeed exclusively is a strong predictor of her actual breastfeeding behavior and positively impacts her chances of success [22, 25, 32].

#### Maternal cognitions

Numerous studies have highlighted concerns about body image and physical changes associated with breastfeeding. Some mothers are concerned about breast sagging, and anxieties about body shape often discourage them from initiating or maintaining exclusive breastfeeding (EBF). Fear of bodily changes acts as a common mental barrier to EBF. Additionally, social and emotional factors contribute to these concerns [18, 21, 31, 34]. Some mothers discontinued exclusive breastfeeding after becoming pregnant again or avoided breastfeeding in public because of shame or stigma [23]. Infant-related issues include reluctance to breastfeed and delayed lactation [24], early or natural discontinuation [23], and frequent crying [25], causing distress and uncertainty, lowering mothers’ confidence in continuing EBF. Mothers’ personal opposition to EBF discouraged its practice, describing it as physically demanding and stressful [18, 22, 23, 35]. Mothers associate the perceived difficulty and time-consuming nature of EBF, along with sleepless nights, with early discontinuation, which discourages continued EBF practice [21, 30, 31, 36].

#### Breastfeeding barriers

Various physical health factors, such as maternal illness [29] and postnatal complications, directly impede exclusive breastfeeding (EBF) by affecting a mother’s capacity to initiate or sustain breastfeeding. In more severe cases, these health issues led to early cessation of EBF or necessitated alternative feeding options, like using wet nurses—particularly in instances of maternal death [32]. Breastfeeding challenges like lactation issues, low milk supply, and feeding difficulties greatly affected exclusive breastfeeding (EBF). A crucial predictor of successful EBF was the ‘absence of lactation problems.’ Nipple pain is a common obstacle that can discourage some mothers from continuing EBF [20, 36]. Poor knowledge of proper breastfeeding technique (positioning and attachment) contributed to lactation issues, affecting mothers’ decisions to start or continue EBF [32]. Actual low milk supply, rather than perceived insufficiency, was identified as a common challenge that hindered mothers from maintaining EBF [21, 30, 36]. Mothers’ worries about infant illness or malnutrition often originated from their personal experiences of inadequate milk supply [32].

## Discussion

The percentage of mothers that practice exclusive breastfeeding from birth to six months across the reviewed studies ranged from 12.5% in humanitarian or rural contexts [32] to 73.8% in urban, facility-based studies [27], with an overall mean of 46.4%; similar to other study and review done in other countries in the sub-Saharan region that reported setting as predictor of Early breastfeeding initiation and EBF rate [41–49]. This reflects some progress toward the WHO 2025 target (50%) but still falls short of the SDG 2030 goal (60%) and the global average of 48% [50]. These variations point not only to methodological diversity but to underlying structural inequities in health infrastructure, socioeconomic conditions, and maternal support systems in Nigeria also to underlying structural inequities in Nigeria’s health infrastructure, socioeconomic conditions, and maternal support systems. Definitional inconsistencies regarding what qualifies as “exclusive” and “early initiation” can further misrepresent breastfeeding levels, reinforcing the need for context-specific interpretations of prevalence data [51].

Maternal age emerged as a recurrent, though inconsistent, predictor. Older mothers (≥30 years) were more likely to maintain EBF, probably due to experience and confidence [24, 29], while younger mothers (20–30 years) also showed favorable practices when supported [21, 36]. A study in Uganda reported similar findings regarding maternal age and EBF rate [47]. However, other studies suggest that age alone cannot determine practice variation, as it often intersects with counselling exposure, work demands, and family roles [18, 24, 29, 35, 36].

Educational qualification also demonstrated mixed influence. While several studies reported that tertiary education correlated with improved EBF knowledge and practice [18, 21, 26, 35], others reported no apparent effect [25], and in a similar study conducted in Cameroon, higher education was associated with earlier cessation [52]. These contradictions suggest that knowledge alone is insufficient without an enabling environment [53–55]. Education may empower mothers but also expose them to competing pressures such as career demands or social acceptance of formula feeding.

Occupational status and employment structure played a nuanced yet influential role in shaping EBF. While formal employment, particularly in education and health sectors, was associated with higher breastfeeding knowledge and uptake [26], rigid schedules and limited maternity leave in formal roles often constrained sustained practice [18, 19, 34]. These constraints were particularly evident among civil servants and professionals who returned to work shortly after childbirth. By contrast, mothers with low incomes in informal roles sometimes maintained better EBF continuity, likely due to greater job flexibility [22], similar to a study in Tanzania that reported a higher EBF rate among housewives than among employed mothers [48]. This suggests that a flexible, supportive work environment is more crucial to a mother’s decision to practice EBF than income alone. It also implies that employment structures such as maternity leave policies have a significant impact on overall breastfeeding behavior.

Marital status and parity showed similarly nuanced effects. While marriage often offered support [36], some dynamics, including early marriage, polygyny, and male-dominated decision-making, hindered mothers from making informed breastfeeding decisions [32]. Parity presented inconsistencies: first-time mothers delayed breastfeeding due to traditional constraints [33], while multiparous women cited fatigue and shifting priorities [32]. Overall, socio-demographic variables should not be interpreted as fixed indicators of breastfeeding behavior. Instead, they often reflect broader systems of support or barriers affecting mothers differently depending on issues like healthcare access, family roles, culture, and gender expectations. Exclusive breastfeeding (EBF) decisions are rarely made by mothers alone but are negotiated within a broader socio-cultural ecosystem involving spouses, elders, extended family members, and wider community expectations. Family support was positively linked to EBF practice [21, 25, 27, 32], while disapproval from partners [22], grandmothers [35], or the absence of supportive networks [23] often undermined maternal intent, revealing the ambivalent nature of social influence. In many settings, elderly women assume authority during postpartum care and infant feeding, similar to findings from other studies in Sub-Saharan Africa [54, 55], highlighting the need to consider influential community dynamics in maternal, newborn, and child health.

Cultural interpretations of infant well-being further shaped feeding behaviors. Perceptions that breastmilk alone is insufficient and infants require water or other feeds [18, 21, 22, 32, 34] are usually due to fears of dehydration, weakness, or sunken fontanels [36], prompting early introduction of fluids or traditional remedies. These beliefs, while medically inaccurate, come from a genuine maternal concern often reflected in context-specific logics in hot climates where infant distress is readily attributed to thirst or hunger. The widespread notion that all living things require water reinforces the perception that EBF is a modern or “Western” practice [56], despite traditional breastfeeding customs often aligning with WHO guidelines. This highlights a knowledge gap regarding biomedical evidence that breast milk comprises 87–88% water and supplies complete nutrition [5]. Also, definitional inconsistencies, such as whether giving plain water disqualifies EBF, complicate cross-cultural comparisons [51]. In many households, traditional knowledge systems retain stronger credibility than biomedical advice.

Traditional healers also shape practices by advising cessation of breastfeeding in cases of maternal illness, HIV, or multiple births [32], while rituals such as infant uvulectomy or postpartum seclusion periods delay initiation and disrupt early feeding [33]. These beliefs surrounding EBF are not merely “barriers” but part of dynamic care systems where tradition, and local logics shape maternal choices. A recurring issue is that most interventions target mothers directly through health facilities, overlooking wider social actors who significantly shape feeding decisions. Without engaging those who hold decision-making power within caregiving networks, improving maternal knowledge alone may not lead to sustained behavioral change. 

The use of health facilities and the support and interactions of health workers positively influenced EBF practice [22], highlighting the importance of consistent breastfeeding support over time [36]. In contrast, visits from lactation experts yielded lower practice rates in one study [25], suggesting that outcomes may vary depending on the study context, other behavioral factors, and culturally appropriate counselling. Proximity to health facilities was associated with higher EBF rates [26], while limited access to functional healthcare facilities was a barrier [32], pointing to the need for adequate and accessible health services, especially for mothers in under-served areas.

Attendance or engagement with antenatal care (ANC), either in a hospital setting [18, 22, 24] or with traditional birth attendants [33], had a significant influence on the timely and early initiation of breastfeeding. This suggests that experiences within the health system shape maternal health behaviors and practices. This is corroborated by more extensive data from Rwanda [57], demonstrated that mothers who gave birth in a hospital had a higher chance of receiving immediate obstetric and postnatal care, as well as breastfeeding education on topics such as feeding benefits, proper positioning, attachment, and breast care, improving mother’s ability to initiate and sustain EBF.

The place and mode of birthing, mostly hospital-based and via vaginal delivery contributed to improved EBF practices and outcomes [18, 29, 30, 33, 36]. This may be linked to the prenatal and postnatal feeding advice that mothers received while at health facilities. On the other hand, mothers’ perception that body pain or complications from caesarean delivery limited skin-to-skin contact and early breastfeeding initiation could lead to delayed lactation and the early introduction of other liquids, ultimately discouraging sustained EBF practices. In addition, health workers during antenatal and postnatal care [21, 27, 35] and mass media [20, 32] were significant sources of breastfeeding information, suggesting combined influence the Socio-Ecological Model (SEM) and the Health Belief Model (HBM) in shaping health behaviors at the individual and community levels.

Maternal knowledge and awareness regarding EBF and colostrum are strong determinants of practice [19, 27, 31, 36]. However, awareness alone does not guarantee adherence, as high awareness did not necessarily translate into practice [18]. This gap stems from a superficial understanding of the term “exclusive breastfeeding” without grasping its full implications [19, 23].

Misconceptions about colostrum often perceived as “bad milk” and discarded, particularly among first-time mothers and in lower-income settings [22, 32, 33], demonstrate how cultural beliefs mediate knowledge. Awareness campaigns must not only convey facts but also address deeply rooted beliefs. These highlight the urgent need for comprehensive, culturally aware breastfeeding instruction that gives mothers confidence and valuable skills, empowering them to overcome barriers and dispel misunderstandings. 

Three reviewed studies provided evidence that targeted interventions such as community and health facility-based support from Médecins Sans Frontières (MSF) in humanitarian contexts [32], Social and Behavior Change programs [22], and drama viewing with prenatal/postnatal follow-up [36], effectively improved EBF outcomes. Despite these examples, a notable gap remains in evaluating breastfeeding interventions in Nigeria.

While a mother’s positive intention often predicts actual practice [25, 32], her motivation and confidence to practice are sustained by self-efficacy [21, 22] supporting the Health Belief Model (HBM) framework which posits that EBF practice, like other health behaviors, is essentially a personal choice [22]. In internally displaced persons settings, the limited availability of health workers to support EBF [32] indicates a contextual environmental influence. Within such contexts, the Socio-Ecological Model (SEM) offers further insight into how multiple levels of influence shape health behaviors, suggesting the need for additional intervention-based research in Nigeria.

However, specific barriers and concerns undermine maternal confidence and sustained EBF, including postnatal illness [29, 32], body shape concerns [21, 31, 34], and fear of breast sagging [18]. These suggest the need for improved maternal care and mental health support to sustain breastfeeding efforts. Also mothers stopped EBF while avoiding breastfeeding in public due to discomfort or being ashamed [23], illustrates infrastructural gaps, as most Nigerian public spaces and organizations lack designated nursing facilities.

Some mothers ceased EBF upon becoming pregnant again [23]. Aside from this, societal stigma is sometimes associated with the myth that breastfeeding during pregnancy negatively affects the nursing child’s development, such as delayed walking or speech or even other social interactions. This highlights a critical knowledge gap within communities and points to the need for clearer public education on infant and child feeding practices within specific social and cultural contexts.

The intensity of the EBF practice itself was a deterrent, reported as stressful [23, 35, 36], intensive [21, 30, 31], and physically demanding [18], suggesting the need for continuous health education and support, facilitative policies to promote EBF practice.

Additionally, breastfeeding challenges such as perceived low milk supply [21, 30, 36], nipple pain [36], and delayed milk onset [24] were commonly reported barriers to sustained EBF. In contrast, the absence of lactation difficulties was significantly associated with higher rates of EBF [20], indicating that these challenges reflect a broader gap in maternal support and practical breastfeeding guidance. For example, inadequate instruction on proper positioning and latching contributed to feeding difficulties [32]. Evidence from studies in India and Denmark [58, 59] reinforces that breastfeeding technique training is essential in preventing early cessation. Similarly, other Nigerian studies [60, 61] emphasize the role of technique in sustaining EBF, yet such support was rarely addressed in the reviewed interventions.

## Strengths and limitations

To the best of our knowledge, this review is the first of its kind to integrate quantitative, qualitative, and mixed studies, as well as studies in developed and less developed areas (rural and humanitarian settings), making it unique in providing an exhaustive insight into the state of EBF in Nigeria. This provides opportunities for general recommendations on intervention and further research to inform policy, practice, and impact.

On the other hand, the review also has some weaknesses. First, it has been conducted by a lone reviewer, which creates the risk for potential researcher bias in the selection of studies and data interpretation. This limitation was mitigated by adopting a self-assessment approach for quality appraisal, which provided a grounded summary and critique of the relevant literature in response to the research questions.

Additionally, the 19 studies reviewed present reports on the six geopolitical zones of Nigeria, which comprise 36 states and the Federal Capital Territory; however, the evidence primarily reflects 15 states and the FCT, with limited data or literature available on the other states. Although all the zones are duly represented, this may affect the generalizability of facts since evidence from other states was not represented.

Other limitations were from the reviewed studies which are listed in the table, and include a lack of in-depth insight based on study designs, issues with generalizability due to study setting, and inconsistent presentation of variables and interpretation for missing or excluded responses in the result tables [19] within the socio-demography and health facility use, for instance, marital status, parity, mode and place of birthing, and ANC and PNC attendance, which are likely to indicate barriers for mothers to practice EBF. Another limitation is that studies had limited data on key EBF practices such as skin-to-skin and early initiation and exclusion of data regarding any EBF health outcomes for both mother and infant and interventions, which reduces the ability to generalize on prevalence rates and factors influencing the mother’s decision to initiate and continue EBF practice.

## Recommendations for policy and practice

To strengthen exclusive breastfeeding (EBF) in Nigeria, policymakers should expand and improve antenatal and postnatal care (ANC/PNC) and community‑based breastfeeding support, particularly in underserved areas such as rural communities and IDP camps.

Additionally, National surveys and health information systems should adopt a single, standardized definition of EBF and early initiation to ensure consistent monitoring and evaluation against WHO and SDG targets.

Furthermore, interventions should be designed to reflect community realities by involving key stakeholders, such as spouses, elders, and traditional leaders, and integrating breastfeeding support into broader family and community systems. Health facilities can complement this by publicizing peer‑mentor schemes, creating private lactation spaces, and training staff in culturally sensitive counselling. Workplace policies must also evolve to support lactating mothers through extended maternity leave, flexible schedules, and breastfeeding-friendly environments.

Finally, the ministries of health and their related partners should invest in robust monitoring and evaluation systems that link EBF practices to maternal-child health outcomes, ensuring that programs are continually refined based on real-world evidence.

## Future research

To address the methodological gaps in the EBF literature in Nigeria, future studies should employ qualitative, mixed-method, and intersectional designs to capture mothers’ in-depth experiences beyond the constraints of Likert-scale surveys, which often fail to capture the complexity of maternal behaviors and challenges. There is a need for further research to consistently collect and report on key variables like skin‑to‑skin contact, early initiation, parity, delivery mode and place, and both antenatal and postnatal care attendance, alongside socio‑demographic and economic indicators such as age, education, occupation, income, religion, ethnicity, and marital status. These approaches will provide a richer understanding of the socio-contextual realities that influence maternal decisions.

Clear operational definitions of “exclusive breastfeeding” and transparent data‑handling procedures (including justifications for exclusions and methods for addressing missing values) are essential to improve comparability and credibility across studies.

Longitudinal and intervention-based studies should be urgently adopted to assess both short- and long-term outcomes of EBF on maternal and child health in Nigeria. Evaluating the effectiveness and scalability of culturally tailored interventions will provide actionable insights for program design. Priority must be given to under‑represented contexts (rural, humanitarian settings, IDP camps) to ensure findings reflect the full diversity of Nigerian mothers. Finally, building national research capacity through training, standardized methodologies, and institutional support will strengthen the quality and policy relevance of future EBF research.

## Conclusion

This review enhances the existing literature by providing a context-sensitive, multi-dimensional analysis of EBF practices in Nigeria. It shows a notable increase in EBF adoption and practice in both urban and rural areas. Despite high awareness levels, systemic and relational barriers hinder the sustained practice of EBF. The decision to start and continue EBF varies, influenced by socio-cultural and maternal factors. The review also points out research gaps, such as limited involvement of fathers, informal caregivers, and mothers in displaced or rural settings. It emphasizes the importance of equity-focused policies and interventions that go beyond individual awareness to address structural factors enabling maternal health and breastfeeding.

## Electronic supplementary material

Below is the link to the electronic supplementary material.

Supplementary Material 1

Supplementary Material 2

## Data Availability

The datasets used and/or analyzed during the current study are available from the corresponding author on reasonable request.

## Abbreviations

ANC: Ante-natal care
BF: Breastfeeding
BFHI: Baby-Friendly Hospital Initiative
CBF: Continued Breastfeeding (up to two years old)
CGs: Caregivers
EI: Early initiation immediately or few hours after birth
EBF: Exclusive breastfeeding
FCT: Federal Capital Territory
HCWs: Health care workers
HF: Health Facility
HV: Hospital visits
HWs: Health Workers
LGA: Local Government Area
NEBF: Not exclusively breastfed
NDHS: Nigerian Demographic and Health Survey
PNC: Postnatal care
PHCs: Primary Health Centers (operate at the LGAs and Community level)
SHCs: Secondary Health Centers (both public and private at the state level)
STSC: Skin-to-skin contact
TBA: Traditional Birth Attendant
THF: Tertiary Health Facility (at the Federal level, e.g. Teaching hospitals)
UNICEF: United Nations Children’s Fund
WHO: World Health Organization
