# Supplementary material for: Exclusive breastfeeding practices and challenges in Nigeria, Sub-Saharan Africa: an integrative review
Source: Int Breastfeed J. 2026 Feb 3;21:26. doi: 10.1186/s13006-026-00810-3 (PMC12958626; doi:10.1186/s13006-026-00810-3)
Supplement: Supplementary file 1 — Supplementary Material 1 [file 13006_2026_810_MOESM1_ESM.pdf]

Reviewed Studies	Aim	Setting/Location (Area, State, Region)	Design/Sample size	Age range, Parity, mode & place of birthing, ANC & PNC attendance.	Education level, Employment & Income Status	EBF Knowledge, Intentions, Practices	Results (Influences, Challenges, Interventions)
Adamu et al. (2022) **[18]**	To determine the prevalence and influencing factors of EBF.	•THF (1), Semi-Urban area.<br>•Sokoto, North-West	•Cross-sectional Descriptive.<br>•Nursing mothers of 6 to 24months infants = 240	• 17 to 45years. •Mostly Primiparous to Multiparous mothers<br>•ANC attendance (98.8%)<br>•Mostly Hospital birthing	•Majority secondary (32%) and tertiary (22.9%) level.<br>•Unemployed (41.3%)<br>•Employees & Self-employed (58.7%)	•EBF practice (34.2%)<br>•Knowledge of EBF benefits (72.2%)	•**Influencing factors:** maternal level of education, occupation, ANC attendance, place of delivery (Hospital).<br>•**Challenges:** limited Knowledge on EBF Important, short maternity leave, breast milk insufficiency, babies require more water, EBF practice is demanding, Fear of breast sagging.
Akadri & Odelola (2020) **[20]**	To determine breastfeeding practices among ANC attendees.	•THF (2), Semi-Urban areas.<br>•Ogun, South-West	•Cross-sectional study.<br>•Parous expectant mothers = 340	•20 to 40years<br>•Primiparous to Multiparous, One or two births (70.9%)<br>•All ANC attendees<br>•Mostly Hospital Birthing	Mostly tertiary level education (50.6%)	•EBF practice (58.8%)<br>•Early initiation (38.8%)<br>•CBF beyond a year (72.4%)	•**EBF Predictors:** Hospital delivery, ANC attendance, information source (Health workers, Mass media), absence of BF issues.<br>•**Challenges:** lactation problems (cracked nipple)
Aliyu et al. (2019) **[19]**	To determine the acceptance of EBF by female health workers.	•THF (1), Urban area.<br>•Kebbi, North-West	•Cross-sectional Descriptive.<br>•Female Medical practitioners = 123	•<20 to 40 years>	Employed between 1 to 15years.	•EBF practice (70.1%)<br>•Awareness (95.1%), and provided correct definition (52.1%)<br>•Knowledge of EBF advantages (30.9%)	•**Influences:** Good knowledge of EBF nutrient such as water in breastmilk.<br>•**Challenges:** Work resumption demands and unfamiliar about EBF practice.

Amat Camacho et al. (2023) **[32]**	To explore caregivers' and health workers' experiences and perceptions of BF practice, promotion, and support for 0 to six months old infants.	•MSF Project Centres, Humanitarian setting (IDP Camp).<br>•Borno, North-East	•Qualitative (Focus groups, interview and observational)<br>•Mothers, Caregivers and Health workers = 32	•20 to 55years<br>•Multiparous (2 to 10 births)	Mostly no formal education.	•EBF practice (12.5%) of 4 women<br>•Positive BF perception.	•**Influences:** Mother's desire and Marital dynamics, Family preference and support, Level of BF Knowlegde, BF promotions and support (Media, Health facilities, NGO, Community level), breastmilk insufficiency, Sampling of EBF healthy infants and fear of illness or malnutrition (infants).<br>•**Challenges:** Maternal health conditions, Knowledge gaps on BF techniques, Perceptions of colostrum as bad, low breastmilk production, traditional beliefs and community practice, Limited BF support workers (Access to HF), inadaptability of support.<br>•**Interventions:** MSF Breastfeeding support (Community and Health facility based).
Anaba et al. (2022) **[22]**	To examine the relationship between women's breastfeeding behaviours and ideations' influences on early initiation and exclusive breastfeeding practices.	•Non-specific wards.<br>•Kebbi, Sokoto & Zamfara, North-West	•Cross-sectional population based.<br>•Women with infants under 2 years = 3039 (with infants 0–5 months old = 721).	•15 to 49years •Mostly birthing outside a health facility.<br>•Majority had No ANC visit up to four times.	•Mostly no formal education (84%).<br>•On-site employment (50.3%).<br>•Mostly Low income household (24%)	•EBF practice (37.5%)<br>•Early initiation (42.1%)<br>•STSC (29.8%)	•**Influences:** Knowledge of BF benefits and practice, Confidence to practice (Self-efficacy), Good perception of Colostrum, Health care providers, Maternal employment and ANC+4 attendance.<br>•**Challenges:** Personal opposition to EBF, Spousal disapproval, Maternal Perception of Colostrum as bad and breastmilk as inadequate or unnecessary.<br>•**Interventions:** Ongoing SBC Programmes
Anazonwu et al. (2018) **[34]**	To explore the attitude and cultural determinants of EBF among childbearing mothers in Nsukka Urban.	•Community (3), Urban area.<br>•Enugu, South-East	•Mixed method (Cross-sectional = 592, In-depth interview = 18).<br>•Mothers, grandmothers & fathers = 610	• 15 to 45 years •Mostly Multiparous 4 children and above (43.5%)	•Mostly secondary education (24.8%).<br>•Employed Civil Servants (34.5%)<br>•Low Income earners	EBF practice (39.9%)	•**Predictors**: Good Personal attitude and perception. Family decision/approval, beneficial outcomes on EBF infantS.<br>•**Barriers**: Work pressure, Family and social support, Body shape or image concerns, Cultural belief on feeding practices (infants needs other food/liquid, male infants should be more breastfed than female).

Anyanwu et al. (2014) **[23]**	To determine the actual breastfeeding practices of HCWs in a tertiary hospital.	•THF (1), Urban area.<br>•Ebonyi, South-East	•Cross-sectional descriptive.<br>•Female Healthcare workers = 100	•21 to 42 years<br>•Multiparous 4 or more children (68%).	Employed between 2 years or more.	EBF practice (25%) for atleast one child, (3% for all children)	•**Barriers to EBF**: Work resumption or busy schedule, EBF too stressful, No knowledge of EBF benefits, Family support, Infants' natural cessation, Expecting another baby, Ashamed to BF.
Atimati & Adam (2020) **[24]**	To determine the breastfeeding practices of mothers of children two years and below.	•Community (1), Semi-Urban area.<br>•Edo, South-South.	•Cross-sectional descriptive.<br>•Mothers = 418	•18 to 50years<br>•Mostly Hospital Birth (82.3%), between 1 and 6 births.<br>•ANC attendees in Hospitals (94.3%)	•Mostly Secondary education level (62.4%).<br>•Self-employed (68.2),<br>•Middle income earners (51.7%)	•EBF prevalence (36.6%)<br>•Early Initiation (44.5%)	•**Predictors of EBF practices**: Maternal education (Tertiary level), Early initiation (ANC attendees at TBA).<br>•**Challenges to EI**: Babies refusal to suck, Delayed lactation.
Balogun et al. (2017) **[30]**	To compare the knowledge, attitude, and practice of breastfeeding among mothers of under-five children.	•Community (2), Urban & Rural area.<br>•Lagos, South-west.	•Comparative cross-sectional study.<br>•Mothers (124 for each area) = 248.	•18 to 55 years.<br>•Mostly Hospital and vaginal delivery.	•Mostly Secondary level and above (Urban 85%, Rural 58.1%)<br>•Employed professionals and skilled workers	•EBF practice = 52.4% (Urban 29%, Rural 75.8%).<br>•Early Initiation = 59.6% (Urban 43.5%, Rural 75.8%)<br>•Positive attitude to EBF (Urban 52.4%, Rural 57.3%).	**Reasons for Non EBF practice:** Mothers' perception (need to feed water), EBF not convienent, low breastmilk poduction, Work resumption.

Bisi-Onyemaechi et al. (2017) **[25]**	To determine the factors affecting knowledge and practice of EBF.	•THF (1), Urban area.<br>•Enugu, South-East.	•Cross-sectional descriptive.<br>•Mothers = 304.	•19 to 50years<br>•ANC attendance (80%)<br>•Hospital birth (69%), Vaginal delivery (88.8%)	•Mostly secondary level and above (97.3%)<br>•Self- employed and formal jobs (72.7%)	•EBF practice (26%)<br>•EBF awareness (98%) and Intentions (75%)	•**Influences:** Intentions/Plans to EBF, Family support.<br>•**Challenges:** Early work resumption, Mother's perception (Breastmilk insufficiency, 'Baby always cries', infant would refuse transition to other feeding)
Elegbua et al. (2023) **[31]**	To compare the impact of knowledge of breastfeeding practices and attitude towards EBF.	•PHC (4), Urban & Rural areas.<br>•Rivers, South-South.	•Comparative cross-sectional.<br>•Nursing mothers (126 for each area) = 252	• 16 to 45 years	•Mostly Secondary level and above (83.4%)<br>•Civil servants and self-employed (75.4%)	•EBF practice = 60.3% (Urban 66.7%, Rural 54%)<br>•Early initiation = 58.4% (Urban 61.9%, Rural 54.8%)<br>•Positive attitude to EBF (Urban 84.1%, Rural 73.8%)	**EBF predictors:** Mother's attitude, awareness and concerns about body shape after BF and Time intensivity of EBF.
Joseph & Earland (2019) **[33]**	To explore the sociocultural factors that influence exclusive breastfeeding among rural mothers.	•LGAs (2), Rural area.<br>•Katsina, North-West.	•Qualitative (Interview).<br>•Mothers = 20.	•18 to 39years<br>•Parous to Multiparous<br>•Home and Vaginal birthing<br>•Minimal ANC visits.	•Mostly no formal education.<br>•Unemployed (Homemakers)	•Low EBF practice and Low early initiation practices. •Adequate EBF knowledge.	•**Predictors:** Family members decisions, HWs and TBAs guidance at ANC visits/place of birthing, Partner's knowledge on BF benefits, Religious beliefs, Early initiation determined by Parity or baby gender.<br>•**Barriers:** Inadequate Family support, Maternal knowledge and perception (Breast milk insufficiency, Colostrum is bad milk), Traditional and Cultural practices (Infant uvulectomy and mothers 40 days bathing).

Mohammed & Aliyu (2021) **[26]**	To determine the knowledge, acceptance, and practice of EBF among caregivers.	•THF (1), Urban area.<br>•Kano, North-West.	•Cross-sectional descriptive.<br>•Mothers & Fathers = 270.	•19 to 60years<br>•Primparous to Multiparous.	•Mostly Secondary (29.3%) and Tertiary level (49.6%).<br>•Employees and Self-employed (66.7%)	•EBF practice (68.5%)<br>•Knowledge of EBF benefit (60%)<br>•Good perception (51.5%)	**Influences:** good knowledge and acceptance of practice, educational level, occupation, and proximity to health facilities.
Odu et al. (2016) **[27]**	To determine the EBF knowledge, attitudes and practices (KAP) of nursing mothers.	•PHCs (5), Urban (4) & Rural (1) areas.<br>•Osun, South-West	•Cross-sectional descriptive.<br>•Nursing mothers = 328	•<20 to 40years>	•Mostly Secondary level (66.5%).<br>•Employees and self-employed (92.7%)	•EBF practice (73.8%).<br>•EI practice (75%)<br>•Good Knowledge of EBF practice and benefits (75%).	**Influences:** adequate knowledge and attitude to EBF practices and benefits, Spousal support.
Ogundairo et al. (2024) **[36]**	To assess the effect of drama intervention on the Breastfeeding self-efficacy (BFSE), initiation, and EBF of pregnant women.	•Community (2), Rural area.<br>•Oyo, South-West.	•Quasi-experimental study (2 groups, control & experimental).<br>•Pregnant women = 200	•<20 to 39years.<br>•Mostly Multiparous (77%).<br>•ANC attendees (73.5%)<br>•Vaginal delivery (76%)	•Mostly secondary level (60%)<br>•Self-employed (74%)<br>•Low income earners.	•EBF practice = 33.1% (Experimental 43.2%, Control 22.9%)<br>•EI practice =58.9% (Experimental 65.5%, Control 52.3%).	•**Predictors:** age, marital status, education, occupation, birthing mode.<br>•**Influences**: Knowledge and awareness of Breastfeeding importance and key practices, pre-natal and post-natal follow-ups, Spousal support.<br>•**Challenges:** Employment type, breastfeeding issues (Nipple pain, low breastmilk production), fear of baby Sunken Fontanel, weight loss, frequent meals, baby being thirsty, and sleepless nights led to early cessation.<br>•**Interventions:** Two weeks Drama viewing before birthing and follow-ups at post-partum.

Okoroiwu et al. (2021) **[28]**	To assess the Knowledge, Attitude and Practice of EBF among mothers.	•PHCs (4), Urban area.<br>•Abuja (FCT), North Central.	•Cross-sectional descriptive.<br>•Mothers = 150	•18 to 50years.<br>•All ANC attendees	No data presented.	•EBF practice (54.4%).<br>•Early initiation of BF (99.32%).<br>•Good knowledge (64.7%)<br>•Positive attitude (70.0%)	**Influences:** Knowledge of BF and Colostrum benefits, Religion, Literacy. Challenges: Illiteracy and Cultural practices.
Olasinde et al. (2021) **[29]**	To assess the determinants of EBF practices among mothers.	•THF (1), Urban area.<br>•Oyo, South-West.	•Cross-sectional descriptive.<br>•Nursing mothers = 271	•<20 to 50years.<br>•Mostly ANC attendees (87.5%)<br>•Multiparous (55.4%)<br>•Hospital birthing (61.3%), Virginal birth (67.5%).	•Mostly post-secondary level (65.3%)<br>•Middle class (79%)	•EBF Prevalence (46.1%).<br>•Early initiation (40.6%)	•**Associating factors:** Mothers' age >30 years, vaginal delivery, and family size >4.<br>•**Determinants:** Family size and birthing mode (vaginal delivery).<br>•**Challenges:** Maternal illness, pressure from relatives to alternate feeding, work resumption.
Ugboaja et al. (2013) **[35]**	To determine the major barriers to postnatal care and exclusive breastfeeding among urban women	•Community markets (4), Semi-Urban area.<br>•Anambra, South-East.	•Mixed Method (Cross-sectional and focus group).<br>•Women = 398.	•20 to 35years><br>•Primiparous to Multiparous (56.8%)<br>•ANC attendees (97.2%)<br>•PNC attendees (91.7%)	•Mostly Secondary level (65.8%), Tertiary level (19.3%).<br>•Self-employed (81.9%)	EBF practice (35.9%)	•**Predictors:** lower maternal age, Higher education level, ANC and PNC attendance (Information source).<br>•**Challenges:** EBF was stressful, Grandmother's refusal.

Yakubu et al. (2023) **[21]**	To assess the knowledge, practice, and factors influencing EBF among nursing mothers.	•THF (1) & SHF (1), Urban area.<br>•Kaduna, North-West.	•Cross-sectional study.<br>•Nursing mothers = 230	•<20 to 50years><br>•ANC and PNC attendees<br>•Hospital birthing (92.6%)	•Mostly Secondary (49.1, Tertiary level (43.9)<br>•Self-employed (53%) , Employees (20%)<br>•Middle income earners (57.8%)	•EBF practice (70%)<br>•Early initiation (69.1%)<br>•Adequate knowledge and awareness (86.5%)	•**Predictors:** lower maternal age, increased educational qualification and good occupation.<br>•**Influences:** Self-motivation, Family and Friends support, information source.<br>•**Barriers:** low breastmilk production, belief that infants require extra fluids, fear of change in body shape or image, cultural practice and societal beliefs, work stress and EBF is intensive.

Limitations
•Study design and use of Likert scale responses fail to capture the full depth of mothers' EBF experiences.<br>•Lacks data on EBF outcomes and the effectiveness of interventions.<br>•No data on key BF practices such as skin-to-skin contact after birth.
•Study method limited insights into breastfeeding practices.<br>•Lacks data on key Breastfeeding practices such as skin-to-skin contact after birth.<br>•No data on the EBF outcomes and any interventions.<br>•Lack of data on employment or income status of participants.
•Likert scale questions may not capture the full depth of mothers' EBF experiences.<br>•No justification given for missing or excluded responses in the result tables.<br>•No data on place and mode of birthing.<br>•Limited generalisability as findings may not apply beyond the study setting.<br>•Lacks data on key Breastfeeding practices such as skin-to-skin contact after birth.<br>•No data on any EBF health outcomes and interventions.

•Results may not be generalisable due study setting.<br>•Lacks data on mode and place of birth.<br>•No data on socio-economic status of participants especially non-health workers and non-IDPs.<br>•Limited data on key BF practices like STSC, and EI.<br>•Limited data on EBF and BF support outcomes from mothers.
•Likert questions may have introduced recall bias.<br>•No operational outline of birthing facilities that respondents used.<br>•No data on mothers' parous status.<br>•Limited data on the effectiveness of interventions on particpants.
•Likert questions may have introduced recall bias.<br>•Limited data on Key BF practices like STSC, EI and adopting EBF.<br>•Lacks data on maternal mode and place of birthing.<br>•No data on EBF health outcomes and any interventions.

•Likert questions may have introduced recall bias.<br>•Study design may not capture the full depth of EBF experiences.<br>•No data on place or mode of birthing and STSC after birth.<br>•Limited generalisability beyond study setting.<br>•No data on any EBF health outcomes and interventions.
•No data on influences to initiate and practice EBF.<br>•Limited data on broader EBF outcomes and any interventions adopted by mothers.<br>•Lacks data on STSC after birth and Early cessation of CBF.<br>•Study design may not capture the full depth of EBF experiences.
•Likert scale questions may have introduced response bias.<br>•No justification for exclusion of some response data.<br>•No data on Socio-demographic factors like Parity, ANC visits and STSC practice at birth.<br>•Lacks data on EBF health outcomes or any interventions adapted.

•Likert scale questions did not capture in-depth responses on mother's experiences.<br>•No data on EI and STSC.<br>•Limited data on EBF health outcomes and any interventions.<br>•No information on Parity of respondents.
•Likert scale questions may have introduced response bias.<br>•Study method did not capture in-depth data on mother's BF experiences.<br>•No data on Socio-demographic factors like Parity, ANC visits, mode and place of birth.<br>•Lacks data on breastfeeding practices like STSC after birth, EBF health outcomes and any interventions.
•Recall bias may have influenced some mothers' responses.<br>•Limited data on key BF practices like STSC, EBF prevalence rate.<br>•Minimal data on EBF health outcomes and interventions.

•Limited data on Mothers' BF barriers and practices like EI, STSC.<br>•Lacks data on EBF health outcomes and impact of interventions.<br>•Minimal data on Socio-demographic factors like ANC visits, mode and place of birth.
•Likert scale questions may have introduced recall bias and limits in-depth data on mother's BF experiences.<br>•Limited data on STSC after delivery, parity, ANC visits, mode and place of births.<br>•Lacks data on mothers' EBF challenges.<br>•Minimal data on EBF health outcomes or any interventions.
•Lacks data on practice of STSC after delivery.<br>•No data on place of delivery.<br>•Minimal data on EBF health outcomes.

•No data on key socio-demographic data like Education level and socio-economic Status of mothers.<br>•Lacks data on Parity, mode & place of birthing and STSC after birth.<br>•Likert scale questions may have limited in-depth data on mother's BF experiences.<br>•Approach to result presentation and analysis limits data interpretation.<br>•Minimal data on EBF health outcomes and effectivenss of interventions.
•Likert scale questions may have introduced recall bias and limits in-depth data on mother's BF experiences.<br>•Minimal data on Employment status and STSC after birth.<br>•Lacks data on EBF health outcomes or any interventions adopted.
•No operational definition for Excusive Breastfeeding.<br>•Likert scale questions may have introduced recall bias and limits in-depth data on mother's BF experiences.<br>•Minimal data on STSC after birth and Early initiation.<br>•No data on mode & place of birthing and STSC after birth.<br>•Lacks data on EBF health outcomes or any interventions adopted.

•Likert scale questions may have introduced recall bias and limits in-depth data on mother's BF experiences.
•No data on mode of birthing and STSC after birth.
•Lacks data on EBF health outcomes or any interventions adopted.
