# Supplementary material for: Exclusive breastfeeding practices and challenges in Nigeria, Sub-Saharan Africa: an integrative review
Source: Int Breastfeed J. 2026 Feb 3;21:26. doi: 10.1186/s13006-026-00810-3 (PMC12958626; doi:10.1186/s13006-026-00810-3)
Supplement: Supplementary file 2 — Supplementary Material 2 [file 13006_2026_810_MOESM2_ESM.pdf]

Study	EBF	Early initiation	STSC
Odu et al. [27]	73.8	75	
Aliyu et al. [19]	70.1		
Yakubu et al. [21]	70		
Mohammed & Aliyu, [26]	68.5		
Elegbua et al. [31]	60.3	58.4	
Akadri & Odelola, [20]	58.8	38.8	
Okoroiwu et al. [28]	54.4	99.3	
Balogun et al. [30]	52.4	59.6	
Olasinde et al. [29]	46.1	40.6	
Anazonwu et al. [34]	39.9		
Anaba et al. [22]	37.5	42.1	29.8
Atimati & Adam, [24]	36.6	44.5	
Ugboaja et al. [35]	35.9		
Adamu et al. [18]	34.2		
Ogundairo et al. [36]	33.1		
Bisi-Onyemaechi et al. [25]	26		
Anyanwu et al. [23]	25		
Amat Camacho et al. [32]	12.5		

Fig. 3

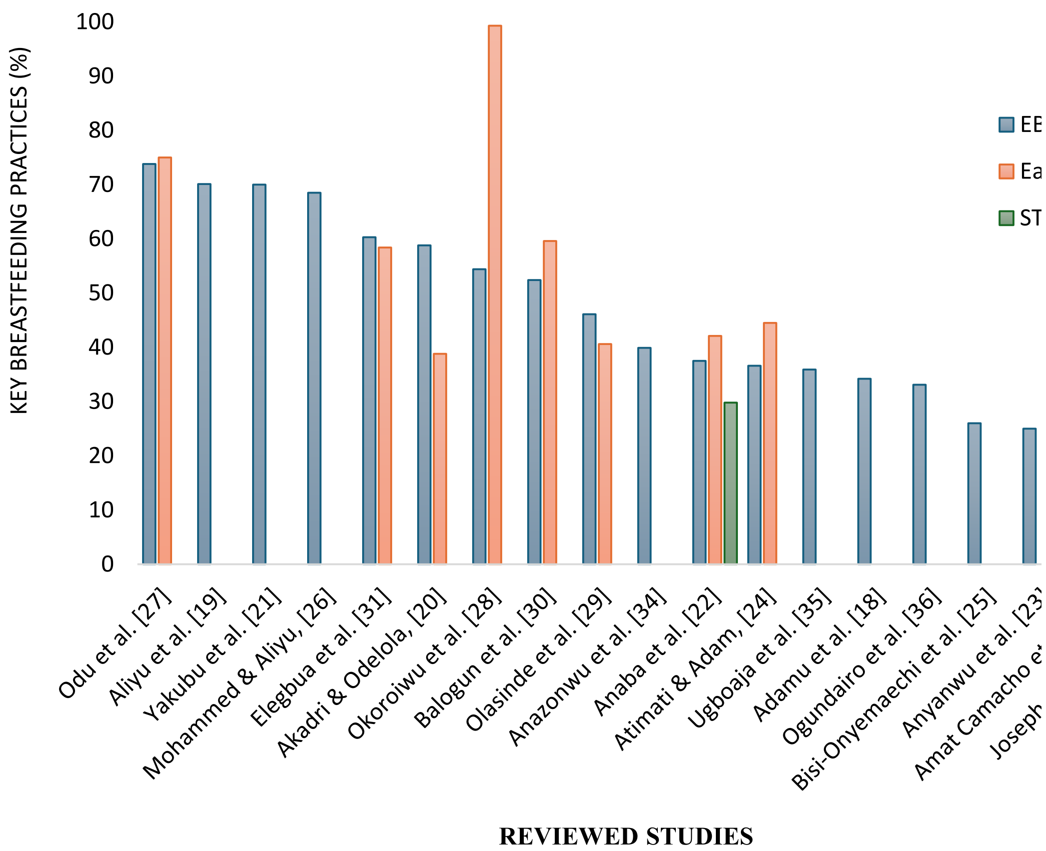

Supplementary Table 2 with Figure 3: Summary of Prevalence Rate of EBF, EI

Joseph & Earland, [33]	Low EBF	Low EI	

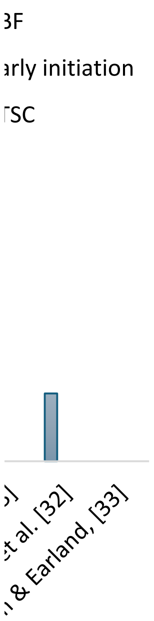

3F
arly initiation
TSC
t al. [32]
& Earland, [33]
